# Is Telomere Length a Biomarker for Aging: Cross-Sectional Evidence from the West of Scotland?

**DOI:** 10.1371/journal.pone.0045166

**Published:** 2012-09-24

**Authors:** Geoff Der, G. David Batty, Michaela Benzeval, Ian J. Deary, Michael J. Green, Liane McGlynn, Alan McIntyre, Tony Robertson, Paul G. Shiels

**Affiliations:** 1 Social & Public Health Sciences Unit, Medical Research Council, Glasgow, United Kingdom; 2 Institute of Cancer Sciences, University of Glasgow, Glasgow, United Kingdom; 3 Centre for Cognitive Ageing and Cognitive Epidemiology, University of Edinburgh, Edinburgh, United Kingdom; 4 Department of Epidemiology & Public Health, University College London, London, United Kingdom; CNRS, Université de Bourgogne, France

## Abstract

**Background:**

The search for biomarkers of aging (BoAs) has been largely unsuccessful to-date and there is widespread skepticism about the prospects of finding any that satisfy the criteria developed by the American Federation of Aging Research. This may be because the criteria are too strict or because a composite measure might be more appropriate. Telomere length has attracted a great deal of attention as a candidate BoA. We investigate whether it meets the criteria to be considered as a single biomarker of aging, and whether it makes a useful contribution to a composite measure.

**Methodology/Principal Findings:**

Using data from a large population based study, we show that telomere length is associated with age, with several measures of physical and cognitive functioning that are related to normal aging, and with three measures of overall health. In the majority of cases, telomere length adds predictive power to that of age, although it was not nearly as good a predictor overall. We used principal components analysis to form two composites from the measures of functioning, one including telomere length and the other not including it. These composite BoAs were better predictors of the health outcomes than chronological age. There was little difference between the two composites.

**Conclusions:**

Telomere length does not satisfy the strict criteria for a BoA, but does add predictive power to that of chronological age. Equivocal results from previous studies might be due to lack of power or the choice of measures examined together with a focus on single biomarkers. Composite biomarkers of aging have the potential to outperform age and should be considered for future research in this area.

## Introduction

Increasing chronological age is associated with a greater prevalence of a wide range of diseases, including most cancers, renal disease, cardiovascular disease and neurodegenerative conditions. At a subclinical level, increasing age is associated with the decline of functional capacity in a number of seemingly disparate areas, including: lung function, muscular strength, arterial flexibility and cognitive ability. However, there is also a great deal of variability between individuals of the same chronological age which has been taken to imply that chronological age is only an imprecise indicator of functional, or biological, age. Others have cautioned against ascribing any causal role to age *per se* [1]. These considerations have prompted the search for more informative markers of aging, referred to as biomarkers of aging.

Baker and Sprott [2] defined a biomarker of aging (BoA) as “a biological parameter of an organism that either alone or in some multivariate composite will, in the absence of disease, better predict functional capacity at some late age than will chronological age”. Despite proffering this definition, they did not believe it likely that a biomarker would be found that satisfied the stronger form of the definition, ie one that would *alone* be a better predictor than chronological age. Their scepticism was later vindicated when the American Federation for Aging Research concluded that 15 years of supported research on BoAs had failed to identify any (see [3]). Johnson's review of progress towards identifying BoAs concluded “the general feeling of the aging community is that biomarkers fulfilling all of the [National Institute on Aging] criteria are unlikely to exist.”

One biomarker that has attracted much attention as a suitable candidate is telomere length. Telomeres are specialized nucleoprotein complexes at the end of eukaryotic chromosomes. They function to cap the chromosome, distinguishing the chromosome end from DNA breaks and thus preventing chromosomal fusions. Telomeres shorten during cell division due to the ‘end replication problem’ [4], [5]. This attrition can be mitigated in germ cells, stem cells and some blood cells via the action of the enzyme telomerase but, because telomerase is essentially inactive in most somatic cells [6], [7], their telomeres are progressively shortened until exhaustion triggers cell senescence. The rate of telomere attrition may also be influenced by levels of oxidative stress [8]. Thus telomere length not only has a clear rationale for being regarded as a BoA at the cellular level, but it also has the potential to explain environmentally induced differences in rates of ageing.

The case for telomere length as a BoA at the level of the human organism was considered in a review by von Zglinicki and Martin-Ruiz [9]. They found evidence of shorter telomeres in affected tissues in several age-related conditions and diseases: immunosenescence, cardiovascular disease, sarcopenia, osteoporosis, osteoarthritis and skin aging. Moreover, there were plausible mechanisms whereby telomere attrition might play a causal role. They concluded that telomere length was a highly promising BoA, whilst at the same time cautioning that it might simply be acting as a proxy for long-term stress rather than a genuine cause.

Mather and colleagues [10] conducted a systematic review of the epidemiological evidence relating telomere length to mortality/longevity and to measures that decline with ‘normal’ aging. They found few mortality studies and those that they did find all suffered from survivorship bias, whereby less healthy contemporaries of the study members have already died before telomere length was ascertained. For associations of telomere length with age related functional decline they found: two studies of lung function, three of grip strength, five of cognitive function and seven of blood pressure. The results for physical functioning were equivocal although lack of statistical power was a serious problem. Stronger support was observed for an association with cognitive function but even there the results were “not unequivocal”. One additional study examined the relationship to self-rated general health and found a significant association.

More recently, Bendix et al [11] studied 548 Danish twins aged 70+ and found an association with self-reported physical ability (strength) but not cognitive function. In contrast, Harris et al [12] studied 1,000 70 year old Scots and found no association with grip strength or lung function but did find an association with cognitive function amongst women.

In this study we provide further evidence from a large, community based, sample – the West of Scotland Twenty-07 study. This study is the same order of magnitude as the two largest studies identified in the review by Mather et al and has comparable measures of physical and cognitive functioning, namely: FEV_1_ as a measure of lung function; grip strength; pulse pressure; four choice reaction time (CRT) and scores on part I of the Alice Heim 4 (AH4) test of general mental ability [13]. We also consider the association of telomere length to three measures of general health, including the same measure of self-rated health used by Njajou et al [14] in the study included in the review by Mather et al.

We also evaluate telomere length against the alternative criterion as part of a composite BoA. This approach requires further elaboration in order to be operationalized. It implies that candidate BoAs need to be identified separately but assessed jointly.

A necessary condition for a BoA to be considered as part of a composite measure might be that it is associated both with chronological age and with one or more age-related measures of functioning or health. The requirement that the measures of functioning be ‘age-related’ implies that they too should be associated with age. So for any given triad consisting of: a candidate BoA, a measure of health or functioning and chronological age, all three pairwise associations would need to be statistically significant.

A sufficient condition for evaluation as part of a composite might be that the biomarker adds explanatory power over and above that of chronological age in the prediction of an age-related measure of functioning or health. This condition would be satisfied if the BoA has a significant association with functioning/health after adjusting for age. If it does, then it clearly has the potential to form part of a composite BoA that outperforms age. A weaker, suggestive, condition would be that the biomarker shares some predictive power with chronological age.

In short, the aim of this study is to augment the epidemiological evidence for telomere length as a BoA with data from a relatively large study, with several measures of health and functioning, evaluating it both as a single BoA and as part of a composite BoA.

## Methods

### Sample

The West of Scotland Twenty-07 Study is a community-based prospective cohort study of 4,510 individuals which was designed to investigate the social processes that produce or maintain inequalities in health and has been described in detail elsewhere [15]. The study consists of three narrow age cohorts recruited at the (approximate) ages of 16, 36 and 56 years in 1987/8 and sampled on a further four occasions over a twenty year period. Respondents who participated at baseline have been shown to be representative of the general population of the sampled area [16]. Analyses are based on the data collected during the final interviews in 2007/2008, when the respondents were aged approximately 36, 56 and 76 years. This was the only occasion on which blood was collected. Data were collected by trained nurses in the homes of the study participants and included blood sampling. Ethical approval for each wave was obtained from the relevant local ethics committee.

### Telomere length

DNA was extracted from peripheral blood leukocytes using the Maxwell® automated purification system according to the manufacturer's instructions (Promega, WI, USA). Telomere length determination was performed blindly using a Roche Light Cycler LC480. Briefly, telomere length analyses were performed in triplicate for each sample, using a single-copy gene amplicon primer set (acidic ribosomal phosphoprotein, 36B4) and a telomere-specific amplicon primer set (27). This method determines the ratio of telomere repeat copy number to single copy gene number (T/S) in experimental samples relative to a control sample DNA. This normalized T/S ratio was used as the estimate of relative telomere length (Relative T/S). Triplicates were averaged prior to further analysis.

### Physical Function

Measures of physical function were lung function, grip strength and pulse pressure. Lung function was measured as the maximum forced expiratory volume in 1 second (FEV_1_) from up to three valid readings on a Micro Medical MS03 spirometer. Grip strength was measured in kilograms using a JAMAR 5030J1 hand dynamometer, and for analyses the mean of up to three readings on the primary hand (highest reading hand if ambidextrous) was used. Blood pressure was measured with an OMRON HEM-705CP automated sphygmomanometer after the respondent had been sitting at rest for 5 minutes. Pulse pressure (systolic – diastolic) is used here.

### Cognitive Function

Two measures of cognitive function are included: Part I of the Alice Heim 4 test of general mental ability [13] and four choice reaction time. Four choice reaction time was measured with a portable battery operated device originally designed for the UK Health and Lifestyle Survey [17]. A description and diagram showing its layout is given by Deary, Der & Ford [18]. The measure used here is the mean choice reaction time of the correct responses out of 40 trials.

### General Health

The measures included here are self-rated health; the number of chronic conditions reported by the participant in response to a standard set of prompts and whether or not the participant is registered disabled. Self-rated health has well documented concurrent and prospective validity [19], [20]. The number of chronic conditions is an indicator for both general levels of morbidity and co-morbidity while registered disability is an indicator of poor physical functioning.

### Statistical Analysis

Linear mixed models were used to relate telomere T/S ratio to age and the measures of functioning in order to control for assay variation by including a random effect of assay plate. Logistic and Poisson mixed models were used for the binary health measures and count of chronic conditions, respectively. Inverse probability weights were used to correct for selective attrition. Principal components analysis was used to form composite measures. To compare the predictive power of age and the two principal component scores, logistic and Poisson regression models were used and the values of the Bayesian Information Criterion (BIC) compared.

As blood was only collected on one occasion all analyses presented here are cross-sectional and based on data from the fifth wave of the Twenty-07 study. The analyses were conducted in SAS version 9.2.

## Results

Of the 2,604 study participants who took part at wave five: 2,568 had personal interviews (as opposed to proxy and postal interviews); 2,310 consented to have telomere analysis performed and blood was collected for 2,242. Sufficient blood was obtained from 2,193 for DNA to be extracted and telomere length was ascertained for 2,186. The mean intra-plate co-efficient of variation for the telomere and 36B4 assays was 0.56% and 0.19% respectively. The amplification efficiency was 95% for both telomere length and 36B4. Respective R^2^ values were .983 and .994. One outlier was subsequently excluded. The working sample comprised those 2,185 with valid measures of telomere length.

Table 1 gives descriptive statistics for the sample broken down by sex and age cohort, labeled by approximate year of birth. Telomere length and the measures of physical function, cognitive function and health all deteriorate with increasing age (for pulse pressure, reaction time, number of chronic conditions and percentage registered disabled, a numerical increase is less favourable). All of these associations with age, including that for telomere length, are significant at p<.001 (see below). Men also had shorter telomeres than women (p = .0246).

**Table 1:** Descriptive information for the study by age cohort and sex.

Mean (SD) N	Women			Men		
	1972	1952	1932	1972	1952	1932
**Age**	36.66	56.96	76.10	36.62	56.99	76.00
	(0.41)	(0.75)	(0.63)	(0.42)	(0.93)	(0.61)
	414	468	310	361	398	234
**Telomere T/S ratio**	0.86	0.79	0.71	0.85	0.77	0.68
	(0.20)	(0.19)	(0.18)	(0.22)	(0.18)	(0.19)
	414	468	310	361	398	234
**FEV_1_ (L)**	2.96	2.27	1.64	3.99	3.23	2.39
	(0.46)	(0.49)	(0.43)	(0.61)	(0.63)	(0.63)
	408	462	296	353	384	223
**Grip (Kg)**	28.15	24.04	18.04	44.87	40.75	31.11
	(5.8)	(6.6)	(6.1)	(9.3)	(8.9)	(7.4)
	394	426	279	343	378	216
**Pulse Pressure (mm)**	41.16	56.02	73.82	48.92	58.10	76.04
	(8.4)	(14.4)	(18.9)	(8.0)	(12.5)	(17.1)
	413	466	304	360	398	234
**Choice RT (ms)**	540.15	635.14	789.61	533.37	634.04	784.05
	(62.2)	(91.1)	(158.0)	(67.8)	(85.4)	(134.3)
	409	458	293	356	393	217
**AH4 score**	38.76	34.54	25.81	40.14	35.91	25.98
	(9.8)	(11.9)	(11.0)	(10.2)	(11.4)	(10.7)
	402	448	265	355	377	206
**Self-rated Health (good)**	75%	69%	53%	80%	74%	58%
	413	466	310	359	397	233
**N of chronic conditions**	1.97	3.02	4.57	1.53	2.64	3.79
	(1.6)	(2.3)	(2.3)	(1.6)	(2.0)	(2.1)
	413	466	310	359	398	233
**Registered disabled**	2%	8%	18%	3%	5%	18%
	413	466	309	358	397	231

Thus, all the measures of health and functioning chosen were significantly ‘age-related’ and, therefore, suitable for testing the extent to which telomere length satisfies the criteria concerning the prediction of functional aging.

**Figure 1:**
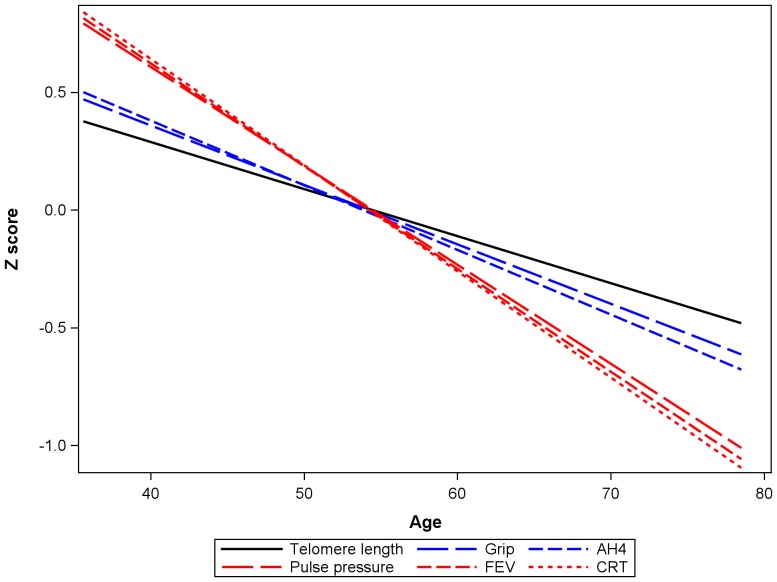
The relationship of telomere T/S ratio and measures of physical and cognitive functioning to age. Lines are fitted values from simple linear regressions on age. All measures are standardized to zero mean and unit SD (z scores) and the sign is reversed for pulse pressure and choice reaction time.

Figure 1 shows the relationship of age to telomere length and to the measures of physical and cognitive functioning. For the purposes of comparison all measures have been standardized to zero mean and unit standard deviation (z scores) and the sign has been reversed for pulse pressure and choice reaction time (CRT) so that lower scores on all variables are unfavourable. Here we see that FEV_1_, pulse pressure and reaction time have the strongest, and very similar, relationships to age. Those for AH4 and grip strength are also very similar but somewhat weaker. Telomere length is slightly weaker again.

Table 2 shows the results of regressing each of the measures of functioning and health on telomere length and age. Results are presented for models where telomere length and age are assessed singly - the model contains *either* telomere length or age - and where they are assessed jointly - the model contains *both* telomere length and age. The final column shows the reduction in the standardized regression coefficients due to mutual adjustment in the joint model. All models are adjusted for sex and assay plate.

**Table 2:** Results of regressing measures of functioning and health on telomere length and age.

	Assessed Singly				Assessed Jointly				
Variable	Estimate	SE	t	p	Estimate	SE	t	p	% reduction
**(a) Telomere Length**									
FEV_1_ (L)	0.259	0.018	14.41	<.001	0.031	0.013	2.28	0.023	88
Grip (Kg)	1.935	0.202	9.59	<.001	0.247	0.187	1.32	0.188	87
Pulse Pressure (mm)	−3.826	0.400	−9.57	<.001	0.772	0.323	2.39	0.017	120
Choice RT (ms)	−48.02	3.315	−14.48	<.001	−7.189	2.636	−2.73	0.006	85
AH4 score	2.702	0.282	9.60	<.001	0.760	0.279	2.73	0.006	72
Self-rated Health (good)	0.259	0.037	7.07	<.001	0.145	0.039	3.73	<.001	44
N chronic conditions	−0.134	0.010	−13.27	<.001	−0.019	0.011	−1.75	0.079	86
Registered disabled	−0.535	0.064	−8.37	<.001	−0.244	0.068	−3.57	<.001	54
**(b) Age**									
FEV_1_ (L)	−0.571	0.012	−48.11	<.001	−0.561	0.013	−44.11	<.001	2
Grip (Kg)	−4.265	0.163	−26.23	<.001	−4.182	0.174	−23.99	<.001	2
Pulse Pressure (mm)	11.076	0.283	39.19	<.001	11.335	0.303	37.46	<.001	−2
Choice RT (ms)	102.96	2.233	46.11	<.001	100.55	2.399	41.92	<.001	2
AH4 score	−5.426	0.245	−22.17	<.001	−5.167	0.261	−19.76	<.001	5
Self-rated Health (good)	−0.361	0.034	−10.52	<.001	−0.314	0.036	−8.62	<.001	13
N chronic conditions	0.314	0.009	33.04	<.001	0.307	0.010	30.33	<.001	2
Registered disabled	0.921	0.066	14.02	<.001	0.844	0.069	12.29	<.001	8

Estimates are per SD of telomere T/S ratio or age.

Results weighted to the baseline sample.

All models include a random effect of telomere assay plate.

Telomere length is significantly associated with each of the measures of health and functioning and in the expected direction: longer telomeres are associated with better health and functioning. Telomere length, therefore, satisfies our necessary condition for a BoA with respect to each of them.

Adjusting for age attenuates the effect estimate for telomere length in every case with most coefficients being reduced by more than 70%. However, the majority of them remain significant. Grip strength and number of chronic conditions were attenuated to non-significance and while pulse pressure has a significant effect in the adjusted model, the effect there is in the opposite direction to that expected. These results reveal two important points. First, the marked attenuation after adjusting for age shows that the majority of the variance predicted by telomere length is shared with age. This is, of course, what would be expected of a BoA. Second, the fact that telomere length remains a significant predictor after adjusting for age in most cases shows that it adds predictive power over and above age.

The results for age show that it is also significantly related to each of the outcomes. This is simply confirmation that the descriptive differences evident in table 1 are statistically significant. Adjusting for telomere length hardly attenuates the effects at all which shows that only a very small part of the age effect is explained by telomere length.

For a BoA to satisfy the stronger criterion proposed by Baker & Sprott it would need to account for a large part, if not all, of the age effect. Clearly, telomere length falls short of achieving this. Nonetheless, since it adds predictive power to that of age, it has the potential to form part of a composite measure which would outperform age as a predictor and would thus satisfy Baker & Sprott's second criterion as a BoA.

Since the measures of physical and cognitive functioning are themselves also candidate BoAs they can be combined into a composite measure with telomere length. It is generally considered that a BoA should reflect “a basic process that underlies the aging process, not the effects of disease” [10]. Consequently, it would be inappropriate to include any of the three health measures.

One simple approach to forming a composite BoA is to take the first unrotated principal component. Two such measures were calculated: the first included the five measures of physical and cognitive functioning and telomere length and the second included the measures of functioning but omitted telomere length. The proportions of the variance accounted for by these principal components were 45% and 53%, respectively. Both principal component scores correlated highly with age at r = −.75 and −.74, respectively. (No importance should be attached to the sign as this is essentially arbitrary.)

Table 3 shows the results of models using age and each of the principal component scores to predict the health measures. For each health measure three separate models are shown: one using age and one each using the two principal component scores as predictors. The Bayesian information criterion (BIC) is used to assess the fit of the models with smaller values indicating better fitting models. For each outcome, both principal component scores produce a better fit than age. However, the very minor difference between the fit of the two implies that telomere length makes only a minor contribution to the overall performance of the composite.

**Table 3:** Results of models using age and principal component scores to predict health outcomes.

Health Measure	Effect	Estimate	SE	t	p	BIC
Self-rated Health (good)	Age	−0.264	0.042	−6.35	<.0001	3750
	PC1	0.631	0.045	13.95	<.0001	3578
	PC2	0.638	0.046	13.94	<.0001	3579
Registered disabled	Age	0.934	0.083	11.30	<.0001	1449
	PC1	−1.182	0.084	−14.14	<.0001	1350
	PC2	−1.178	0.084	−14.07	<.0001	1355
N of Chronic Conditions	Age	0.309	0.011	27.55	<.0001	12635
	PC1	−0.319	0.011	−29.17	<.0001	12573
	PC2	−0.320	0.011	−29.07	<.0001	12583

PC1 includes telomere length; PC2 excludes it.

Based on N = 1867 with complete data for all measures.

## Discussion

In this relatively large, population based, study telomere length was related to sex, age and eight measures of health and functioning. These comprised: three measures of physical functioning (lung function, grip strength and pulse pressure); two measures of cognitive functioning (four choice reaction time and a general mental ability test score); and three measures of health (self-rated health, registered disability, and the number of chronic conditions). As all the measures of health and functioning were themselves related to age, telomere length satisfies our necessary condition for a candidate BoA with respect to all eight measures.

When adjusted for age, telomere length remained significant in a majority of cases demonstrating that it adds predictive power over and above age in five out of eight cases. On the other hand, it only accounted for a very small proportion of the effect of age and hence does not itself satisfy the stronger criterion of being by itself a better predictor than age. This is, perhaps, unsurprising given that two decades of research have not yet resulted in one candidate BoA which does satisfy this criterion and many doubt whether one will be found.

Even though they fall short of the stricter criterion for a BoA the results here offer more support for telomere length as a candidate BoA than the epidemiological evidence to date. There are a number of reasons why this might be the case. First is the issue of sample size and consequent statistical power to detect associations. In the review of Mather et al only two studies had over 2,000 participants, one further study had around 1,000 (and Harris et al [12], published subsequently, also had around 1,000) but the remainder were smaller often considerably smaller. A second reason is the measure(s) of functioning examined. Most studies only examined a single measure – notable exceptions being those of Harris [12], [21] and Mather [22]. The most frequently studied outcome, blood pressure, was the subject of two out of the three largest studies but is one of the weaker associations in this study. The largest study [23] examined cognitive function and did find a significant association. A third issue is the age range covered. Most of the studies had narrow age ranges and two involved birth cohorts [12], [21]. This range restriction will have weakened the observed associations. It is possible that these studies would not even have satisfied our necessary condition of having functional measures that were significantly associated with age. Finally, because of the emphasis on the stronger criterion of a BoA whereby a single measure should outperform age, the studies generally reported results that were adjusted for age. This corresponds to our sufficient condition for consideration as part of a composite BoA. Examining the association in birth cohorts [12], [21] is equivalent to adjusting for age. Taking all these considerations together it is perhaps unsurprising that the evidence to date has been equivocal.

At this point, it is worth mentioning a major limitation both to this and previous studies, namely, the lack of longitudinal data on telomere length. Longitudinal measures have the potential to measure telomere *loss* more accurately than inter-individual comparisons of telomere *length* and this may be a fruitful direction for future research.

Nonetheless, in view of the results to-date and of the widespread scepticism that a single BoA could be found we also evaluated telomere length against the alternative criterion suggested by Baker and Sprott as part of a composite BoA. All the measures of physical and cognitive functioning included here change with normal aging and have themselves been considered as candidate BoAs. Hence they were included in the composites. Principal components analysis was used to construct the composites as it is a relatively simple approach which has the advantage of not relying on any particular theory of measurement or on assumptions about the structure of the underlying dimensions but can be regarded as a simple rotation of the data. We produced two composites from the functioning measures, one with and one without telomere, length which we evaluated against chronological age in the prediction of the health measures. Each produced a better fit than age, although the contribution of telomere length was modest.

There is, however, a major caveat to the interpretation of this result which relates to the purpose for which BoAs are sought, specifically whether they are for the prediction or explanation of biological aging. From the perspective of explaining biological aging, the relatively modest contribution of telomere length to the composite BoA here could be misleading as it operates at a lower level than the other components, ie at the cellular level as opposed to the systemic level. If, for example, telomere attrition was part of the causal process generating age-related functional decline at the higher level, then adjusting for those higher level measures of functioning would underestimate its effect.

Where the aim is purely prediction, the results here suggest that telomere length has relatively little to offer over and above the other measures considered here. This may have important practical and policy implications. As von Zglinicki recently noted [24], telomere length tests are now available commercially and are being actively marketed at the medical profession. Any physicians contemplating this option would be well advised to consider some of the functional measures examined here, such as grip strength, FEV and reaction time. These are all simpler, cheaper (or even free [25]) and have established predictive capacity.

Future research on BoAs should focus more on seeking out composite markers rather than merely pitting candidates against each other [26]. The methods used to do this will vary depending on whether the aim is prediction or explanation and whether a general purpose measure is sought or one optimized to a specific outcome, such as age itself or some measure of health or functioning. One class of statistical techniques suitable for explanatory models are the so called structural equation models within which composite measures are operationalized as latent variables and may be simultaneously related to antecedents and/or outcomes. Where the primary concern is with prediction, machine learning methods might prove more fruitful. In either case, the combination of biologically plausible biomarkers with multivariate statistical methods offers an avenue for progress.
